# Design of Ultra-Narrow Band Graphene Refractive Index Sensor

**DOI:** 10.3390/s22176483

**Published:** 2022-08-28

**Authors:** Qianyi Shangguan, Zihao Chen, Hua Yang, Shubo Cheng, Wenxing Yang, Zao Yi, Xianwen Wu, Shifa Wang, Yougen Yi, Pinghui Wu

**Affiliations:** 1 School of Physics and Optoelectronic Engineering, Yangtze University, Jingzhou 434023, China; 2 State Key Laboratory of Electronic Thin Film and Integrated Devices, School of Electronic Science and Engineering, University of Electronic Science and Technology of China, Chengdu 610054, China; 3 State Key Laboratory of Advanced Processing and Recycling of Non-Ferrous Metals, Lanzhou University of Technology, Lanzhou 730050, China; 4 Joint Laboratory for Extreme Conditions Matter Properties, Southwest University of Science and Technology, Mianyang 621010, China; 5 School of Chemistry and Chemical Engineering, Jishou University, Jishou 416000, China; 6 School of Electronic and Information Engineering, Chongqing Three Gorges University, Chongqing 404000, China; 7 College of Physics and Electronics, Central South University, Changsha 410083, China; 8 Fujian Provincial Key Laboratory for Advanced Micro-Nano Photonics Technology and Devices, Quanzhou Normal University, Quanzhou 362000, China

**Keywords:** graphene, ultra-narrow band, refractive index sensor, terahertz waves

## Abstract

The paper proposes an ultra-narrow band graphene refractive index sensor, consisting of a patterned graphene layer on the top, a dielectric layer of SiO_2_ in the middle, and a bottom Au layer. The absorption sensor achieves the absorption efficiency of 99.41% and 99.22% at 5.664 THz and 8.062 THz, with the absorption bandwidths 0.0171 THz and 0.0152 THz, respectively. Compared with noble metal absorbers, our graphene absorber can achieve tunability by adjusting the Fermi level and relaxation time of the graphene layer with the geometry of the absorber unchanged, which greatly saves the manufacturing cost. The results show that the sensor has the properties of polarization-independence and large-angle insensitivity due to the symmetric structure. In addition, the practical application of testing the content of hemoglobin biomolecules was conducted, the frequency of first resonance mode shows a shift of 0.017 THz, and the second resonance mode has a shift of 0.016 THz, demonstrating the good frequency sensitivity of our sensor. The S (sensitivities) of the sensor were calculated at 875 GHz/RIU and 775 GHz/RIU, and quality factors FOM (Figure of Merit) are 26.51 and 18.90, respectively; and the minimum limit of detection is 0.04. By comparing with previous similar sensors, our sensor has better sensing performance, which can be applied to photon detection in the terahertz band, biochemical sensing, and other fields.

## 1. Introduction

Surface plasmons (SPs) are two-dimensional plane waves propagating along the interface between metal and dielectric, which can confine subwavelength of the electric field in the direction perpendicular to the dielectric for the purpose of controlling light [1,2]. Surface plasmon resonance (SPR), as an embranchment of SPs, is excited by the coupling of photon-electron resonance when the wave vector of the incident light matches that of the surface plasmon wave [3]. The resonance frequency can be tuned by changing the geometric parameters and material of the metal layer, etc [4]. SPR-based biosensors are popular research topics in recent years and have been playing an important role in biological diagnosis and environmental detection on account of its high sensitivity and real-time response [5,6,7,8]. For example, one of the most common applications of SPR sensors is the detection and characterization of different biochemicals, including antibodies and other proteins [9,10]. The conventional SPR sensor is a three-layer dielectric structure proposed by Kretschmann, where a metal film is usually attached to the beveled edge of the prism to adsorb biological or chemical molecules [11]. Since the resonant frequencies of metals must be in the visible spectral range, gold or silver is generally chosen as the material for metal thin films. However, both materials have some defects that cannot be improved (for example, silver is easily oxidized, reducing performance and life of devices; the absorption capacity of biomolecules on gold is poor, and the sensitivity and performance of sensors is limited), thus limiting the effective use of the sensing performance of the devices [12,13]. Therefore, it is essential to find a new material to enhance the sensing performance.

Electromagnetic metamaterial is a new type of artificially designed composite material with a structural size smaller than the wavelength of external incidence, which has peculiar optical properties such as a negative refractive index and a negative magnetic permeability. In addition, the desired metamaterial properties can be achieved by designing subwavelength structures [14,15]. Currently, one of the most promising types of metamaterials for application is graphene, which is a lattice material composed of hexagonal carbon atoms. Graphene has excellent optical characteristic such as high optical transparency, strong electrical conductivity, and strong biosorption due to its special electronic structure [16,17,18,19,20]. It has been found that the optical characteristics of graphene change significantly on the SPR curve, and the graphene increases the sensitivity of the device to changes in the refractive index compared to conventional metallic materials [21]. On the other hand, similar to metals, graphene can support the propagation of surface plasma waves in the mid-infrared and terahertz bands [22]. However, unlike conventional metal SPR, the plasma of graphene is tunable and exhibits dynamic tunability with the method of adjusting the Fermi level and relaxation time of graphene by electrostatic or doping [23,24,25,26]. Using the feature, the actual manufacturing cost of the device is greatly saved, and the device performance can be tuned more easily and quickly.

Based on the advantages of the above properties of graphene materials, it is possible to achieve optimization of sensor performance. In real life, optical sensors of graphene-based SPR can be used for bio-detection such as single cells, antigen antibodies, proteins and so on [27,28,29]. In recent years, a wide variety of graphene absorption sensors with different properties have been proposed. However, most of these absorbers are single-frequency absorbers with complex fabrication steps and poor performance in sensing detection [30,31,32,33]. Therefore, the emergence of a sensor with a simple configuration, dual-frequency absorption and high refractive index sensitivity is an inevitable trend.

Terahertz waves lie between 0.1 and 10 THz, and are mainly excited by intramolecular and intermolecular vibrations [34,35]. Although the terahertz wave band has not yet been fully explored in the electromagnetic spectrum, it has now shown great potential for applications in communication, security, medical, and military, and is of great research value [36,37,38,39]. Actually, there has been some research progress in the combination of graphene absorption sensors and terahertz waves in recent years, but the majority of these works are only one resonance mode or do not achieve perfect absorption [40,41,42], hindering the expansion of application ranges of devices. Based on this, a novel ultra-narrow band graphene THz absorption sensor structure is designed in this paper. The absorption efficiency of the absorber is first calculated by simulation, and its intrinsic electric field distribution and impedance matching principle are analyzed. Then the effects of the Fermi level, relaxation time, polarization angle, and incident angle on absorption are discussed separately. Next, the sensing performance is analyzed and compared with similar ultra-narrowband absorber structures. Finally, the sensor capability is investigated for the detection of biomolecules in the biomedical field. The results show that the designed sensor has dynamic tunability, polarization-independence, large-angle insensitivity, and good sensing characteristics.

## 2. Model Structure

**Figure 1:**
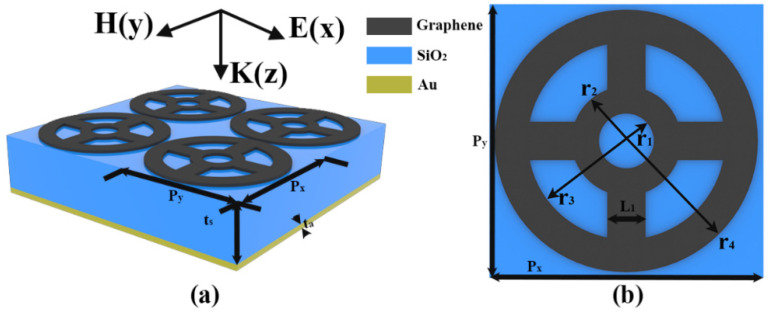
(**a**) Three-dimensional view of the ultra-narrowband graphene SPR absorber array (**b**) Platform of the absorber.

Our proposed ultra-narrowband absorber structure consists of a patterned graphene layer on the top, a dielectric layer of SiO_2_ in the middle, and a bottom metal layer, as shown in Figure 1. The chosen dielectric SiO_2_ has a relative permittivity of *ε_d_* = 1.4 and a thickness of *t_s_* = 28 µm. The structural period of the basic cell is *P* = *P_x_ = P_y_* = 15 µm. The bottom metal layer adopts lossy Au with conductivity *σ* = 4.09 × 10^7^ S/m and the ply t_a_ = 0.5 µm, which can block the transmission of terahertz waves efficiently [43,44]. The inner ring radii *r_1_* and *r_2_* of the top patterned graphene are 1 µm and 3.5 µm, respectively, and the outer ring radii *r_3_* and *r_4_* are 5 µm and 7 µm, respectively. Based on this structure, the simulation was conducted by using FDTD (Finite difference time domain) solutions software [45]. During the process, in *x*- and *y*-directions, periodic boundary conditions are used. In the *z*- direction, perfect matching layer (PML) 24 layers is applied. The simulation temperature in our work is set to 300 K. In the simulations of this paper, the thickness of the monolayer graphene is set to 1 nm. By modulating the material parameters of the graphene layer, it was found that the optimal absorption efficiency of this absorber in the terahertz band was achieved when *E_F_* = 0.7 eV and *τ* = 0.7 Ps.

The total conductivity of graphene we used can be obtained from σ_g_ = σ_intra_ + σ_inter_, with σ_intra_ represents intra-band conductivity, σ_inter_ is inter-band conductivity. According to Kubo formula, the conductivity of graphene can be described by [46,47]:

(1) $$\sigma_{\mathrm{intra}}=\frac{{\mathrm{ie}}^{2}k_{B}T}{\pi \hbar^{2}(w+i\tau^{-1})}\left\{\frac{E_{F}}{K_{B}T}+2\ln\left[\exp(-\frac{E_{F}}{K_{B}T})+1\right]\right\}$$

(2) $$\sigma_{\mathrm{inter}}=\frac{{\mathrm{ie}}^{2}}{4\pi \hbar^{2}}\ln\left[\frac{2\left|E_{F}\right|-\hbar (w+i\tau^{-1})}{2\left|E_{F}\right|+\hbar (w+i\tau^{-1})}\right]$$

where the charge of electron *e* = 1.6 × 10^−19^ C, *K_B_* refers to the Boltzmann constant, *ħ* represents the approximate Planck constant, *T*, *ω* refers to the ambient temperature and angular frequency of the incident wave, respectively. *E*_F_ and *τ* refer to the Fermi level and relaxation time of the graphene layer, respectively. The σ_inter_ of graphene is negligible since *E*_F_ >> *ħω* in the terahertz band, and the surface conductivity of graphene depends mainly on intra-band contribution. Therefore, the total conductivity of graphene can be simplified as Drude formula [48]:

(3) $$\sigma \left(\omega \right)=\frac{ie^{2}\left|E_{F}\right|}{\pi \hbar^{2}\left(\omega +i\tau^{-1}\right)}$$

From the above equation, it is clear that the graphene optoelectronic devices can achieve active adjustability by means of regulating the Fermi level and relaxation time. The property simplifies the design of optoelectronic devices and increases the flexibility in different cases.

## 3. Results and Analysis

**Figure 2:**
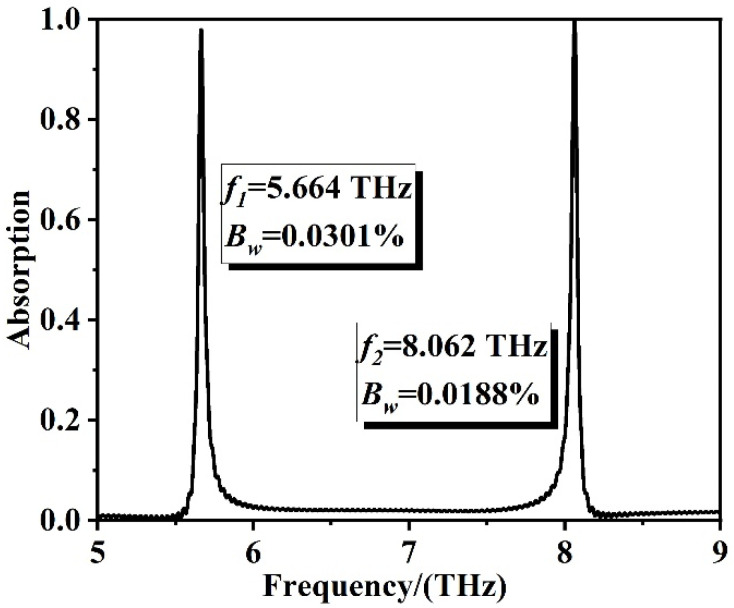
Absorption spectra of ultra-narrow graphene absorber in the range of 5~9 THz with resonance frequency *f* and absorption bandwidth *B_w_* marked on the graph.

As displayed in Figure 2, the patterned graphene absorber achieves ultra-narrow band perfect absorption in the incident frequency ranges of 5~9 THz, and absorption efficiency of 99.41% and 99.22% are achieved at 5.664 THz and 8.062 THz, respectively. And these results were calculated and simulated by 3D-finite difference time domain method in FDTD software. The Q-factors of two resonant frequencies, defined as Q = f_0_/Δf [49], are 171.64 and 196.63, respectively.

To calibrate the bandwidth level of the absorber, the parameter relative absorption bandwidth *B_w_* is used, which is expressed as the ratio of total bandwidth to center frequency, and defined as [50]:

(4) $$B_{w}=2\times \frac{\left(f_{\max}-f_{\min}\right)}{\left(f_{\max}+f_{\min}\right)}\times 100\%$$

where *f_max_* and *f_min_* are the highest and lowest frequencies, respectively. If *B_w_* is less than 1%, it is considered narrowband. If *B_w_* is in the range of 1~25%, it is considered wideband, and if *B_w_* is greater than 25%, it is considered ultra-wide band. In our work, the absorption bandwidths of the two resonant frequencies where the absorption efficiency remains above 80% are 0.0171 (5.6552~5.6723 THz) and 0.0152 (8.0551~8.0703 THz), respectively. Therefore, according to equation (4), the relative absorption bandwidths *B_w_* at the two resonant frequencies were calculated to be 0.0301% and 0.0188%, respectively. *B_w_* is much less than 1%, so the absorber is ultra-narrow band absorption. The total absorption bandwidths are 0.033 THz and 0.041 THz, respectively.

**Figure 3:**
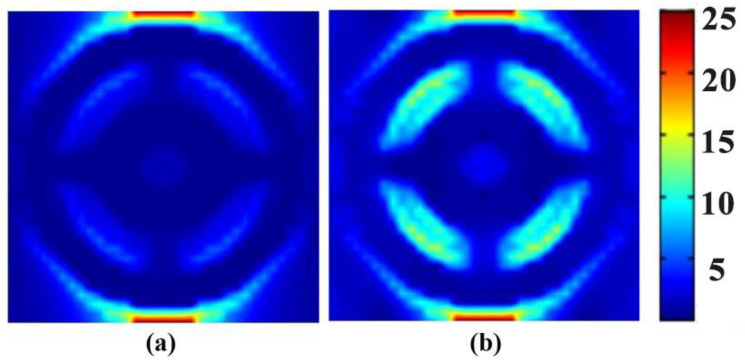
The cross-sectional electric field distribution on the absorber surface in the x-y direction when the incident light frequency is (**a**) 5.664 THz and (**b**) 8.062 THz, respectively.

To investigate the intrinsic mechanism of perfect absorption of the absorber, we set separate frequency-domain field monitors at 5.664 THz and 8.062 THz respectively in *x-y* plane first, then observed and plotted the cross-sectional electric field distribution diagram, as demonstrated in Figure 3. It is worth noting that the electric field we calculated was normalized, different colors represented different intensities of electric field, and the electric field became stronger and stronger from blue to red. The intensity values of electric field corresponding to different colors is presented in the color bar of the electric field. Obviously, the electric field distribution patterns at the two perfect absorption bands were different. For the electric field at 5.664 THz, it was mainly distributed at the upper and lower sides of the outer ring. And at 8.062 THz, not only the graphene SPR of outer ring excited an electric field, but also the inner ring contributed the electric field component. It can be attributed to the coupling of the vibrational frequency of the patterned graphene layer with the terahertz waves in these two frequency bands and providing electric dipole resonance, forming different resonance modes that greatly consumed the energy of the incident light, and the ultra-narrow graphene absorber achieved a perfect match with the free-space impedance in the two resonance frequency bands, finally realizing the perfect absorption of the absorber.

The impedance matching principle is a significant theoretical basis to achieve perfect absorption of the absorber. The equivalent impedance *Z* can be calculated by the Equation (5) [51]:

(5) $$Z=\sqrt{\frac{{\left(1+S_{11}\right)}^{2}-S_{21}^{2}}{{\left(1-S_{11}\right)}^{2}-S_{21}^{2}}}$$

**Figure 4:**
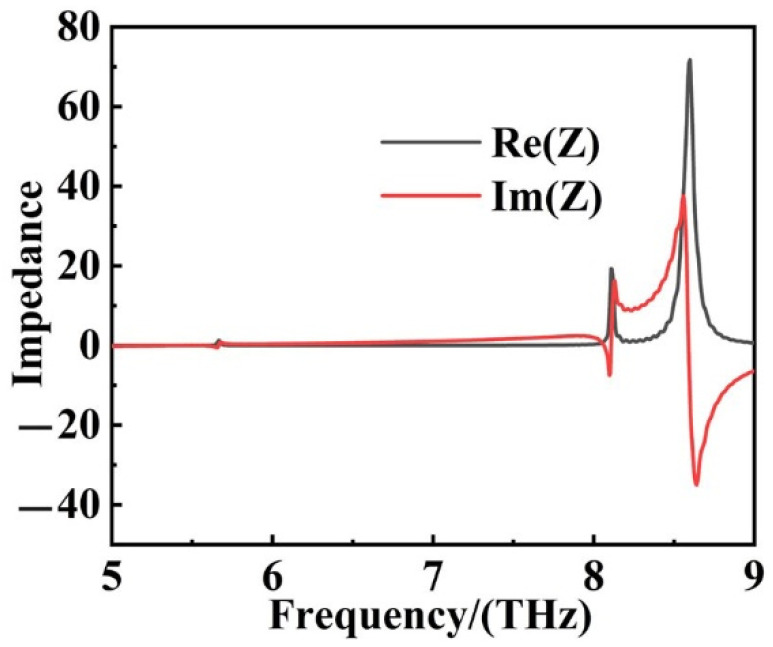
The real part Re(Z) and the imaginary part Im(Z) of the effective impedance Z of the ultra-narrow SPR absorber.

Here, *S*_11_ and *S*_21_ were the scattering parameters related to the reflectance and transmittance, respectively. Derived from the effective impedance matching theory, we could obtain the equivalent impedance *Z* of the absorber from the simulation results, as suggested in Figure 4. When the effective impedance *Z* of the absorber matched with the free space, i.e., the real part (Re(Z)) of the effective impedance *Z* of the system had a value close to 1, and the imaginary part (Im(Z)) was close to 0, so the reflection (*S*_11_ = 0) could be greatly decreased, for which a perfect absorption was acquired. According to Figure 4 and combined with the absorption spectra, it could be found that the absorber achieved a perfect match with the free-space impedance at the resonance wavelengths of 5.664 THz and 8.062 THz, and obtained 99.41% and 99.22% perfect absorption, respectively. The values of real parts of impedance at the two absorption peaks were 0.042 and 0.087, the values of the imaginary part of impedance were 2.16 and −0.096. The resonance around 8.5 THz in the absorption response was because that the impedance we discussed was effective impedance, which was different from impedance. When the Re(Z) and Im(Z) of the effective impedance *Z* deviated from 1 and 0, respectively, the absorption efficiency decreased sharply. It proved that the proposed graphene SPR ultra-narrow perfect absorption was due to the impedance matching at the frequencies of 5.664 THz and 8.062 THz.

**Figure 5:**
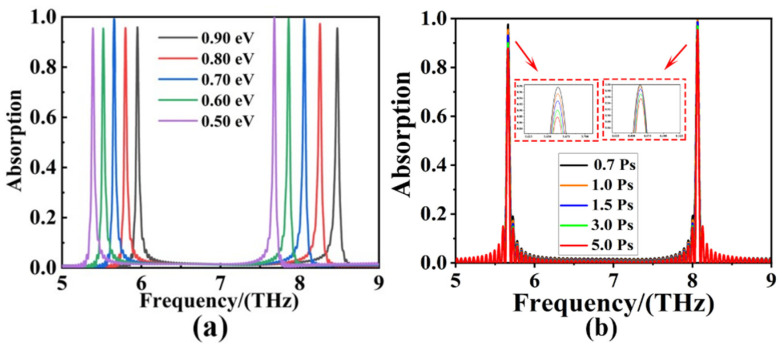
(**a**) Absorption changing spectra with the Fermi level of graphene increased from 0.50 eV to 0.90 eV. (**b**) Absorption spectra obtained by increasing the relaxation time of graphene from 0.70 Ps to 5.0 Ps.

Based on the tunability of graphene materials, the changes of absorption spectra of the absorbers were next investigated by regulating the Fermi level and relaxation time of the graphene layers, respectively, as shown in Figure 5. The equation for the external voltage regulation of graphene Fermi level *E_F_* is as follows [52,53]:

(6) $$E_{F}=V_{F}\sqrt{\pi \varepsilon_{0}\varepsilon_{r}V_{g}/e_{0}t_{s}}$$

where *V_g_*, *e*_0_, *V_F_* and *t_s_* is external voltage, electron charge, Fermi velocity and the ply of SiO_2_ layer, respectively. Among them, *V_g_* can be modulated by adjusting the external voltage or chemical doping. Besides, *ε*_0_ and *ε_r_* denotes the vacuum permittivity and relative permittivity, respectively. Figure 5a demonstrates the blue shift of both absorption peaks of the absorber as the Fermi level incremented from 0.50 eV to 0.9 eV, and the modulation ranges of the resonant frequencies are 5.389~5.951 THz and 7.680~8.474 THz with modulation depths of 0.562 THz and 0.794 THz, respectively. The optimal absorption efficiency is achieved as *E_F_* = 0.7 eV.

The electron relaxation time *τ* of the patterned graphene layer was calculated by [48]:

(7) $$\tau {=E}_{F}v/\left(ev_{F}^{2}\right)$$

where *E_F_*, *ν* is the Fermi level, carrier mobility of graphene, respectively. *e* is the electronic charge, and *V_F_* = 10^6^ m/s. The absorption spectra of the absorber illustrated in Figure 5b as relaxation time *τ* increased from 0.7 Ps to 5 Ps. The results showed that the absorption efficiency changed gradually and the resonance frequency remained unchanged. Another interesting phenomenon that appears in Figure 5b is the significant fluctuations around the two absorption peaks, which can be attributed to the variation of relaxation time of graphene. The carrier’s plasmonic oscillations can be enhanced with the increases of *τ*, and the strong plasmonic oscillations will interact with the surrounding medium, resulting in fluctuations around the two absorption peaks. The modulation ranges of the absorption efficiency were 87.83% to 99.41% and 95.45% to 99.22%, and the modulation depths were 11.58% and 3.77%, respectively. Therefore, graphene absorbers could achieve the tunability of the absorption spectrum by regulating the Fermi level and relaxation time of the graphene layer with the geometry of absorber unchanged, which had a higher value than conventional metal absorbers in more actual fields.

**Figure 6:**
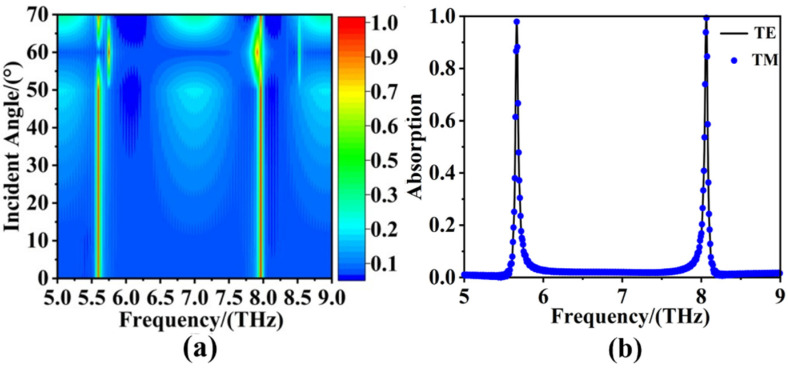
With the incident angle of the source increasing from 0° to 70°, (**a**) the sweep spectra of the absorber under TE and TM polarization. (**b**) the fitted spectrograms of TE and TM polarization.

In real life, vertical incidence plane wave was just one of these cases. The real situation was more complicated and volatile. Therefore, the studies on the insensitivity to oblique incidence of absorber were necessary [54,55,56,57]. Based on this, the variation of the sweep spectra of the absorber under TE (Transverse Electric) polarization and TM (Transverse Magnetic) polarization by changing the incident angle from 0° to 70° were investigated. The TM polarization and TE polarization were defined in terms of whether the electric or magnetic field only had a transverse component. The electromagnetic waves were propagating along the *z*- axis, when the electric field only had a horizontal component in the *x-y* plane, it was called TE waves. When the magnetic field only had a horizontal component in the *x-y* plane, it was called TM waves [58]. Figure 6a is the sweep spectra of the absorber under TE and TM polarization with the incident angle of the source increasing from 0° to 70°. The results revealed that when the incident angle was in the range of 0°−70°, the absorption of TE polarization and TM polarization was the same, i.e., the absorber has the polarization-independent property, and a similar conclusion can also be obtained from the fitted spectrograms of TE and TM in Figure 6b. In addition, the phenomena in Figure 6a also manifest that the ultra-narrow absorber was insensitive to the incidence angle in the ranges of 0° to 70°.

**Figure 7:**
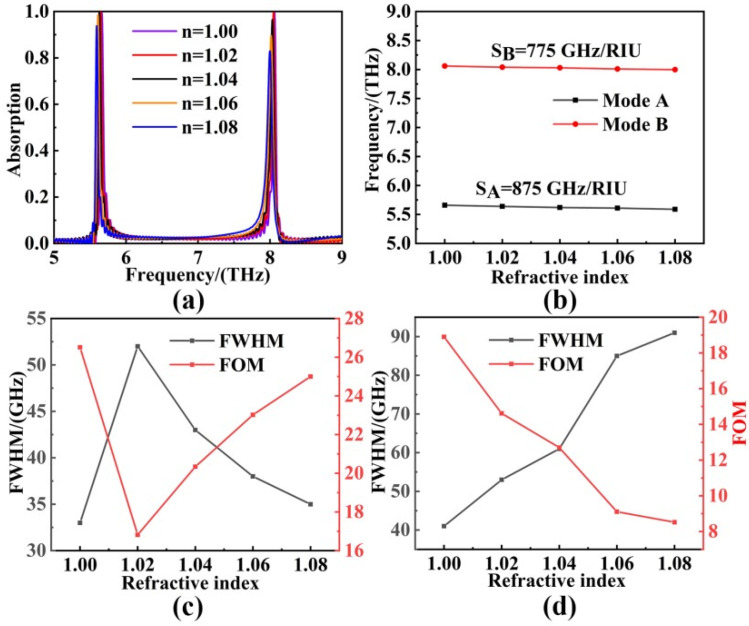
With the surrounding refractive index *n* increasing from 1.00 to 1.08, (**a**) the absorption changing curves of our absorber; (**b**) the *n-f* line chart of two modes; (**c**) The FWHM and FOM are calculated for the first resonant frequency; (**d**) The FWHM and FOM are calculated for the second resonant frequency.

The sensor capability is explored in Figure 7. The curves in Figure 7a suggest that the frequency bands of the two resonance modes were blue-shifted and the absorption efficiency decreased as *n* increased, which, indicating the resonance modes, were sensitive to the refractive index. We then measured the sensor capability quantitatively by calculating the parameters of S (Sensitivity) and FOM (Figure of Merit). According to the sensitivity Formula (8) [59,60]:

(8) S=Δf/Δn

where ∆*f* and ∆*n* are the changes in resonance frequency and ambient refractive index, respectively. Figure 7b fits the sensitivity calculated for the two resonance frequencies of this absorber, and the sensitivity at mode A (at 5.664 THz) and mode B (at 8.062 THz) were 875 GHz/RIU and 775 GHz/RIU, respectively. Then, the FOM of our sensor was obtained from Formula (9) [61,62,63]:

(9) FOM=S/FWHM

where S (Sensitivity) had been given above, and FWHM (Full Width at Half Maximum) was the full width of the half-peak at the resonance frequency. The value of FWHM represents the peaks’ width in the position of the half of absorption efficiency, and can be calculated according to the simulation data. Figure 7c,d calculate FWHM and FOM at the two resonance frequencies, respectively, and these results show that the maximum FOM of mode A = 26.51 and the maximum FOM of mode B = 18.90. In addition, the detection factor

(10) P=FWHM/S

is introduced to assess the sensing performance of our sensor quantitatively since the limit of detection (LOD) is proportional to FWHM/S [64]. And according to formula (10), the calculated detection factors *P* of the two resonance modes were 0.04, 0.05, respectively. The smaller detection factor exhibited a higher refractive index sensitivity and better sensing characteristics of our sensor. After comparing with the works of those who came before us, our absorption sensor had the advantages of dual-band absorption, dynamic tunability, high refractive index sensitivity, and good sensing performance, as shown in Table 1 [65,66,67,68]. The results demonstrated that the absorber had better sensing performance and broader application prospects. The results demonstrated that the absorber had better sensing performance and broader application prospects.

**Figure 8:**
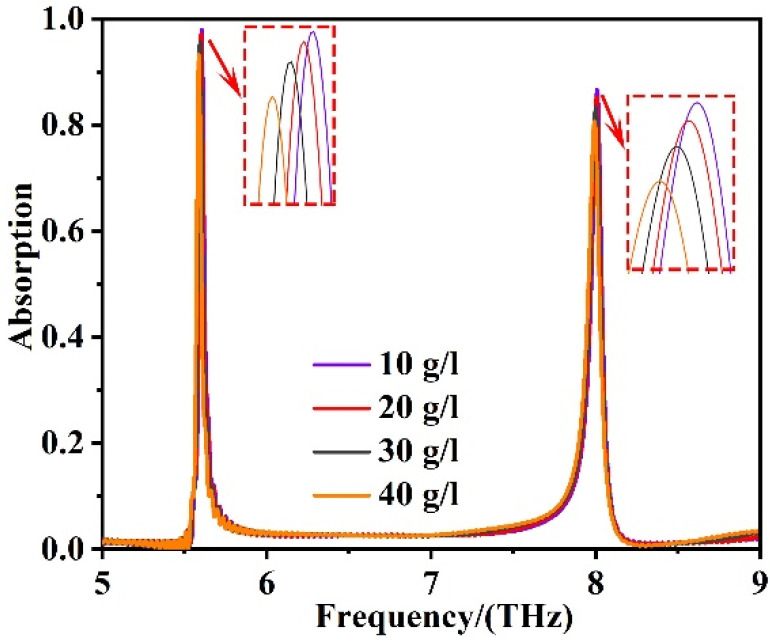
The wavelength changed at the two resonance frequencies when the hemoglobin molecular content increased successively from 10 g/L (n = 1.34), 20 g/L (n = 1.36), 30 g/L (n = 1.39) to 40 g/L (n = 1.43).

Finally, we investigated the sensing performance of our absorption sensor applied in real time. Figure 8 suggests the changing curves when the sensor was designed to measure the content of hemoglobin molecules in organisms [69]. The functionalization of the sensing surface was adsorption. Meanwhile, the problem of nonspecific adsorption was considered. When detecting hemoglobin molecules with our sensor, modifying the sensor with anti-protein nonspecific adsorption material was very significant. The material could effectively prevent nonspecific adsorption of protein on the surface of the device, so as to improve the compatibility of our sensors. Commonly used anti-protein nonspecific adsorption materials are PEG, PEG derivative, and polysaccharide, etc [70]. The shift of frequency is a sign of refractive index changes. And when our sensor detected materials, a different content of hemoglobin molecules can cause different frequency offset, showing the different refractive index of materials. Then, we can find the corresponding content of the hemoglobin molecule by consulting the refractive index libraries of substance. Thus, different content of hemoglobin molecules can be determined. When the content of hemoglobin biomolecules increases successively from 10 g/L (n = 1.34), 20 g/L (n = 1.36), 30 g/L (n = 1.39) to 40 g/L (n = 1.43), the two resonance modes both show a blue shift. The resonance frequencies of first resonance mode shifts from 5.604 THz to 5.587 THz, and the resonance frequencies of second resonance mode shifts from 8.009 THz to 7.993 THz. Compared with the former works, for example, Pang et al. experimentally designed a sensing strategy for specific recognition of hemoglobin with the limit of detection (LOD) as low as 2 [71]. Our sensor achieved the minimum limit of detection of 0.04. These phenomena prove that the sensing system we developed had good sensing performance in specific applications, and it is expected to be applied in more practical fields.

## 4. Conclusions

In this paper, ultra-narrow perfect absorbers in the 5–9 THz band were obtained based on the single-layer graphene SPR structure. By designing the structure, perfect absorption was obtained at 5.664 THz and 8.062 THz with absorption efficiencies of 99.41% and 99.22% and absorption bandwidths of 0.0171 THz and 0.0152 THz, respectively. The relative absorption bandwidths *B_w_* at the two resonant frequencies were calculated to be 0.0301% and 0.0188%, and the Q-factors were 171.64 and 196.63, respectively. Associating with the dynamic tunability of graphene, the resonant frequency bands can be modulated efficiently by adjusting the Fermi level and relaxation time of the top graphene. The polarization-independence and wide-angle insensitivity characteristics of the absorber were studied by changing the polarization mode and incidence angle of the incident light. Finally, the sensing characteristics of the absorption sensor were investigated. The calculated sensitivities of the sensor were 875 GHz/RIU and 775 GHz/RIU, quality factors FOM (Figure of Merit) were 26.51 and 18.90, and the minimum limit of detection was 0.04. In addition, the practical application of testing the content of hemoglobin biomolecules was conducted, and the results show that our sensor had good sensing performance, which can be expected to be applied in optical detection, medical imaging, biosensing, and other fields.

## Figures and Tables

**Table 1:** Comparison of various performance of similar absorption sensors.

References	[65]	[66]	[67]	[68]	Presented
Resonance mode	One	Two	Three	Two	Two
Wave band	0.508 THz	0.4–0.8 THz	1–2.4 THz	23–36 THz	5–9 THz
Couple mode	Guided Resonance	EIT-like	Plasmon	PIT	Plasmon
Tunability	No	No	Yes	Yes	Yes
Sensitivity (GHz/RIU)	23.08	96.2	152.5	26.6	875
FOM(1/RIU)	~	7.8	4.26	~	26.51

## Data Availability

Publicly available datasets were analyzed in this study. This data can be found here: [https://www.lumerical.com/] (accessed on 1 January 2020).
